# Exploring the potential of deep brain stimulation in managing cluster headache: a systematic review

**DOI:** 10.1186/s12883-025-04373-4

**Published:** 2025-08-27

**Authors:** Olivier Uwishema, Sanobar Shariff, Judy Ahmad El Chakik, Lydia Daniel Bisetegn, Omar Alomari, Magda Wojtara

**Affiliations:** 1 Department of Research and Education, Oli Health Magazine Organization, Research, and Education, Kigali, Rwanda; 2 Yerevan State Medical University, Yerevan, Armenia; 3 Faculty of Medicine, American University of Beirut, Beirut, Lebanon; 4 School of Pharmacy, Faculty of Natural Sciences, University of the Western Cape, Cape Town, South Africa; 5 Hamidiye International School of Medicine, University of Health Sciences, Istanbul, Turkey; 6 University of California, Los Angeles David Geffen School of Medicine, Los Angeles, USA

**Keywords:** Cluster headache, Deep brain stimulation, Hypothalamus, Treatment, Neurological disorder, Headache management

## Abstract

**Background:**

The debilitating nature of cluster headache (CH) is typified by intense, repeated cephalgia that are often referred to as “suicide headaches” owing to their severity. Individuals may not respond appropriately to therapeutic interventions despite various alternatives being readily available. This warrants an investigation into further management options. One of the promising solutions for CH has been identified as deep brain stimulation (DBS), which specifically targets the posterior hypothalamus.

**Aim:**

This systematic review aims to summarize the current literature on the safety and efficacy of DBS in the management of CH.

**Method and materials:**

A comprehensive literature search identified 15 relevant studies comprising clinical trials, case reports, and observational studies from Embase, Web of Science, PubMed/MEDLINE, Scopus, and the Cochrane Library using the Preferred Reporting Items for Systematic Reviews and Meta-Analysis (PRISMA) 2020 standards.

**Results:**

The systematic review elucidated that DBS, with a focus on the posterior hypothalamus, facilitated significant reductions in the frequency, severity, and duration of cephalgic ‘attacks’ associated with CH observed. While individual responses varied, DBS was generally well-tolerated, with transient and reversible adverse effects involving focal neurological deficits (i.e., diplopia) being the most common. Importantly, long-term benefits were observed in vast populations, improving their overall quality of life. Furthermore, DBS demonstrated effects beyond localized hypothalamic stimulation, with evidence suggesting modulation of the pain processing network. Additionally, DBS was found to alleviate nocturnal CH attacks without disrupting circadian rhythms. These findings suggest that DBS holds promise as a therapeutic option for CH, particularly in individuals refractory to other treatments.

**Conclusion:**

Existing research suggests that DBS, particularly when targeting the posterior hypothalamus, can significantly reduce CH ‘attack’ frequency, severity, and duration. Further research should focus on patient selection criteria and a deeper understanding of the mechanisms underpinning DBS to optimize its effectiveness in managing such a disabling cephalgia.

## Introduction

Cluster headache (CH) is a neurological disorder characterized by severe, unilateral pain often described as one of the most intense forms of headache management challenges. of immense debilitation affecting thousands of individuals globally. Patient populations exhibit a predominance of males who are more likely to develop CH than females at ages 10 to 50 years old. Particularly, the frequency is observed to be higher when familial in origin [1]. This type of headache is distinguished by persistent, severe headaches that precede in clusters or cyclical patterns. These “attacks” may occur several times daily, normally lasting between 15 min and two hours [2]. The discomfort experienced, which classically concentrates around the eye or temple unilaterally, is frequently described as a stabbing or burning feeling owing to its neuropathic nature. Other symptoms include conjunctival injection, lacrimation, nasal congestion, and increased agitation [3].

Due to its severity, CH is frequently called “suicide headaches” and may have a negative influence on a person’s quality of life [4]. Such “attacks” may result in physical and psychological distress, interrupting activities of daily life and often leaving individuals with substantial disabilities [5]. CH may be managed with different medical or conservative therapies, including pharmacotherapy targeting CVH pathophysiology (such as the use of triptans, corticosteroids, or verapamil), oxygen therapy, and nerve blocks [6]. Although these therapies provide some comfort, many patients do not report their long-term benefits and may experience minimal relief [3], open to alternative practices.

As CH is circadian in nature, some researchers have hypothesized that symptoms may be attributable to a sleep disorder [7]. It is evident, nonetheless, that CH is a brain disorder with a primary disease locus centered on the hypothalamus [8]. Acute CH episodes have been associated with activation of the posterior region of the hypothalamus in numerous studies, ranging from early H_2_O-Positron Emission Tomography (PET) studies to more current research circumventing functional magnetic resonance imaging (fMRI). Additionally, the posterior hypothalamus is activated by trigeminal nociceptive input [9], which in this case appears to be an adequate target for CH treatment.

Recently, Deep Brain Stimulation (DBS) has gained attention as a possible advancement in the treatment of CH [10]. DBS entails inserting electrodes into brain regions indicated—typically the hypothalamus—and attaching said electrodes to a pacemaker-like pulse generator device [11]. With the application of electrical impulses, this device modifies neuronal activity in the desired brain region. DBS remains static in the experimental stage of CH management. However, some studies have demonstrated promising outcomes, including a reduction in cephalgia ‘attack’ frequency, severity, and duration [10, 12]. Functional neuromodulation using DBS has been reported to alleviate the significant pain that is experienced by chronic CH and works by targeting the neurophysiological substrates that mediate pain [13].

Thus, we conducted a systematic review to compile the data supporting DBS intervention as an effective therapeutic for CH since it is imperative to discover novel treatment methodologies. This research has the potential to influence care disseminated for populations affected by CH and provide hope for alternatives to individuals unable to obtain adequate relief.

## Materials & methods

A systematic review was conducted where complete literature retrieval was garnered across several academic databases to explore scientific articles relevant to the outcomes of utilizing DBS in the management of CH. This systematic review followed the Preferred Reporting Items for Systematic Reviews and Meta-Analysis (PRISMA) 2020 standards, guaranteeing a robust and uniform methodology [14]. We submitted the research protocol for this systematic review to the International Prospective Registry of Systematic Reviews (PROSPERO) database (www.crd.york.ac.uk/prospero/) and subsequently was assigned the PROSPERO ID: 2022 CRD42022322666. All working group members agreed on the study protocol before the literature search.

### Search strategy

The electronic search yielded results from five databases: Embase, Web of Science, PubMed/MEDLINE, Scopus, and the Cochrane Library. In August 2023, a search was performed applying a strategic combination of relevant terms with Boolean operators to ensure effective results. The search strategy comprised the following terms: (DBS OR “Deep Brain Stimulations” OR “Brain Stimulation” OR “Electrical Stimulation of the Brain”) AND (Hypothalamus OR “Preoptico-Hypothalamic Area” OR “Lamina Terminalis”) AND (Ventral tegmental DBS OR VTA DBS) AND (Cluster Headaches OR “Ciliary Neuralgia” OR “Cluster Headache Syndrome” OR “Histamine Cephalgia” OR “Horton Syndrome” OR “Horton’s Syndrome” OR “Chronic Cluster Headache” OR “Episodic Cluster Headache” OR “Atypical Cluster Headache” OR “Red migraine” OR “Horton’s headache” OR Cephalalgia OR “Sphenopalatine neuralgia”).

The retrieved studies were carefully assessed based on their titles and abstracts, and only those that met the eligibility criteria were considered for the following stages. The published work was not subject to any time limitations or filters. Additionally, we conducted a manual search by reviewing the references of the included articles and literature reviews for possible relevant studies.

### Study selection

The collected articles were checked for potential duplicates. Following the removal of duplicates, the titles and abstracts were screened by four independent reviewers (J.A.E.C, L.D.B, O.A, and M.W). Any discrepancies between the reviewers were resolved by carefully re-examining the article and discussing it until an agreement was achieved.

**Fig. 1:**
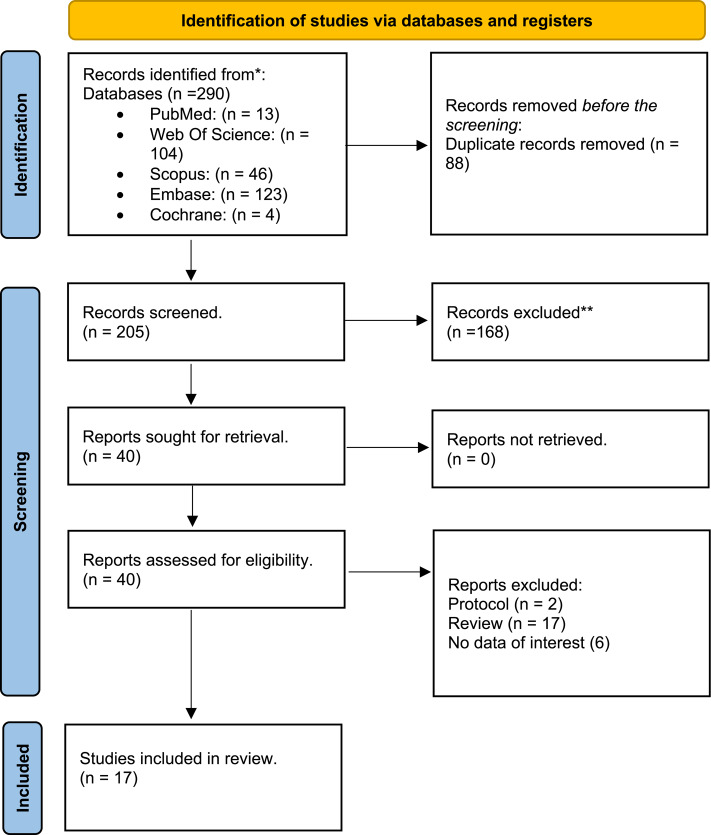
PRISMA 2020 flow diagram for new systematic reviews which included searches of databases and registers only. From: Page MJ, McKenzie JE, Bossuyt PM, Boutron I, Hoffmann TC, Mulrow CD, et al. The PRISMA 2020 statement: an updated guideline for reporting systematic reviews. BMJ 2021;372:n71. 10.1136/bmj.n71

Studies deemed relevant and any conflicts were subjected to full-text screening. Five reviewers (J.A.E.C, L.D.B, O.A, S.S and M.W) obtained the full texts of studies that may have met the criteria. They independently assessed them to decide if they should be included in the final analysis. The process for resolving disagreements during the full-text screening was similar to that used previously. If a resolution could not be reached, a leading author and supervisor (**O.U**) was available to re-evaluate the distinctions and make a final decision regarding whether to include the study in the analysis. The detailed search process and the selection of studies are elaborated in the accompanying PRISMA diagram (see Fig. 1).

### Eligibility criteria

This review focused on patients who had undergone DBS as a therapeutic intervention for CH. The selection criteria were deliberately designed to encompass a wide array of research perspectives, with no restrictions based on language medium or study type. Inclusion criteria encompass all types of relevant studies, including observational studies (such as prospective or retrospective cohort studies, case-control studies, cross-sectional studies, case series, or case studies) and experimental studies (including randomized controlled trials).

To maintain the rigor and significance of the gathered studies, a careful exclusion process has been applied. Duplicate publications, review articles, editorial correspondence, book chapters, animal studies, and laboratory experiments have been excluded. Additionally, studies focused solely on in vitro inquiries have been omitted. This meticulous approach ensures that the studies included in the review meet stringent standards of quality and relevance, contributing to a comprehensive and meaningful examination of the application of DBS in the management of CCH among patients.

### Data extraction

Five authors, (O.U, J.A.E.C, L.D.B, O.A, S.S and M.W) were responsible for conducting primary data extraction, entering the collected information into a pre-piloted Microsoft Excel spreadsheet. To guarantee data accuracy and consistency, a leading author and supervisor (O.U) meticulously reviewed the completed extraction sheet, reconciled any discrepancies, and validated the precision of the data. The extracted information included details about the authors, publication years, study types, sample sizes, targeted area of the hypothalamus, indications for DBS, post-DBS outcome, duration of the procedure, reported adverse events and the main findings expressed within included studies.

### Critical appraisal tool and risk of bias assessment

Two authors (O.A and L.D.B) assessed the risk of bias in the eligible included studies, comprising two relevant clinical trials, and 13 observational studies. The National Institutes of Health (NIH) Quality Assessment Tool for Observational Cohort and Cross-sectional Studies was employed for this purpose [15]. The assessment involved rating studies on a scale of 0 for poor (0–4 out of 14 questions), for fair (5–10 out of 14 questions), and for good (11–14 out of 14 questions). In cases where certain questions were not applicable (NA) or not reported (NR), these designations were used accordingly. The methodological quality of the included clinical trial was assessed by using the RoB2 tool [16]. Any discrepancies in their assessments were resolved through discussions among the researchers until a consensus was reached.

## Results

### Summary of included studies

**Fig. 2:**
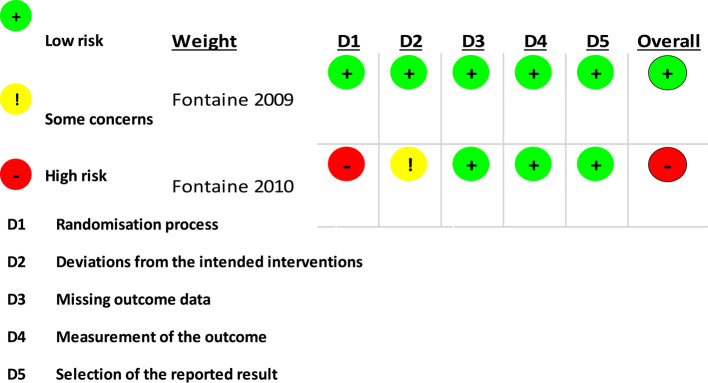
The Results of the RoB2 (Risk of Bias 2) Cochrane Tool Assessment for the Included Randomized Trial Conducted across Five Key Domains

Following the inclusion, exclusion, and deletion of duplicates, 15 articles were included in the final study. This systematic review identified several types of published studies comprising clinical trials and retrospective studies alongside case reports and series. Table 1 summarizes the relevant findings for each included study as well as its characteristics. This systematic review identified studies pertaining to the safety and effectiveness of hypothalamic DBS in managing chronic headaches as well as reporting novel advancements. The overall quality of studies included in the review ranged from fair to good, and no study exhibited poor methodology or was designated as having a high risk of bias. A comprehensive breakdown of the quality assessment results is provided in (Table 2 and 3) and Fig. 2.

**Table 1:** Relevant findings extracted from the included study

Author-year	Title	Study Design	Sample size	Targeted Area of the Hypothalamus	Summary	Adverse Effects
Akram et al. 2017 ^[17]^	Optimal Deep Brain Stimulation Site and Target Connectivity for Chronic Cluster Headache	Observational Study	7	Hypothalamic gray	• 7 patients. • DBS - significant reduction in headache load (76%), attack severity (46%), frequency (58%), and duration (51%) over an average follow-up of 34 months. • The optimal target- near the third ventricle, along the trigeminohypothalamic tract, connecting pain-related brain regions. • No sample size for this research.	Transient dizziness, nausea, intermittent diplopia
Akram et al.2016 ^[18]^	Ventral tegmental area deep brain stimulation for refractory chronic cluster headache	Prospective case series	21	Posterior hypothalamic region	• The study used deep brain stimulation (DBS) targeting the **posterior hypothalamus region near the ventral tegmental area (VTA)**. The **median follow-up** in the study was **18 months** • This area was chosen because it’s implicated in **pain and autonomic regulation** seen in cluster headaches.	Transient and mild side effects reported
Bartsch et al. 2008^[19]^	Hypothalamic Deep Brain Stimulation for Cluster Headache: Experience from a New Multicase Series	Comparative Study	6	The posterior hypothalamus	• 6 patients. • DBS- posterior hypothalamus Four patients experienced significant relief within 6 months, while two had limited improvement. • 17-month follow-up • Three patients were nearly attack-free, and DBS was well-tolerated with no long-term side effects. • No sample size specified.	Transient and mild side effects reported
Cheema et al., 2023 ^[20]^	Association of Clinical and Neuroanatomic Factors With Response to Ventral Tegmental Area DBS in Chronic Cluster Headache	Prospective observational cohort study	43	Ventral tegmental area (VTA)	• All patients had a **minimum of 1 year follow-up** • **Mean follow-up** across the cohort was **5.6 years**	No adverse effects reported
Dantas et al. 2019 ^[21]^	Deep Brain Stimulation Modulates Hypothalamic-Brainstem Fibers in Cluster Headache: Case Report	Case Report	1	The posterior hypothalamus	• One patient • Hypothalamic DBS • The study successfully treated a patient using DBS, and they found that the stimulation contact was in a white matter area behind the mammillary bodies. • Important fiber tracts affected included the medial forebrain bundle and connections between the hypothalamus and brainstem, possibly explaining some of DBS’s clinical effects in CH **treatment**.	No adverse effects reported
Fontaine et al. 2010 ^[22]^	Anatomical Location of Effective Deep Brain Stimulation Electrodes in Chronic Cluster Headache	Prospective Controlled Trial	10	The posterior hypothalamus	• 5 patients • DBS - posterior hypothalamus The effective DBS contacts were located behind the hypothalamus, near various brain structures. • Notably, there were no significant differences in contact coordinates or nearby structures between responders and non-responders, suggesting that treatment outcomes might be unrelated to electrode placement. • DBS could potentially modulate local headache generators or non-specific pain-regulating systems.	No adverse effects reported
Fontaine et al. 2009 ^[23]^	Safety and Efficacy of Deep Brain Stimulation in Refractory Cluster Headache: A Randomized Placebo-Controlled Double-Blind Trial Followed by a 1-Year Open Extension	RCT	11	The posterior hypothalamus	• In a study involving patients with severe, refractory CCH, DBS targeting the hypothalamus was assessed. During a randomized phase, comparing active and sham stimulation for one-month periods, no significant differences were observed. However, during the subsequent one-year open phase, more than 50% of patients responded to chronic stimulation, with some becoming pain-free. • Three serious adverse events occurred, but no significant changes in hormonal functions or electrolyte balance were noted. • While the randomized phase didn’t show DBS efficacy, the open phase suggested long-term benefits for CCH treatment, warranting further controlled studies.	Serious adverse events reported-subcutaneous infection, transient loss of consciousness and micturition syncope.
Franzini et al. 2003 ^[24]^	Stimulation of the Posterior Hypothalamus for Treatment of Chronic Intractable Cluster Headaches: First Reported Series	Case Series	5	Posterior hypothalamus	• 5 patients • In the treatment of drug-resistant chronic cluster headaches, DBS of the posterior hypothalamus was effective and safe. • All five patients remained pain-free for 2 to 22 months after treatment, with some not needing medication. • There were no side effects, complications, or tolerance issues observed, indicating the potential of posterior hypothalamic DBS for this condition.	No adverse effects reported
Franzini et al. 2004 ^[25]^	Hypothalamic Deep Brain Stimulation for the Treatment of Chronic Cluster Headaches: A Series Report	Prospective Cohort	8	Posterior hypothalamus	• 8 patients • This study introduced a new surgical treatment for drug-resistant chronic CH. Eight patients received chronic high-frequency stimulation of the posterior hypothalamus, resulting in improvements for all of them. • Some patients became pain-free without medication, and no harmful side effects or complications were observed. These preliminary results suggest that hypothalamic stimulation is safe and effective for treating drug-resistant chronic CH, affirming the central role of the hypothalamus in the condition’s pathogenesis. • All of the patients reported being pain-free at 1–26 months of follow-up.	No adverse effects reported
Franzini et al. 2008 ^[26]^	Neuromodulation in Treatment of Refractory Headaches	Clinical Case	9	Posterior hypothalamus	• Neuromodulation is emerging as a promising option for drug-resistant conditions like CCH and trigeminal neuralgia. • This report discusses patients who underwent DBS of the Posterior Hypothalamus for these conditions, with positive clinical outcomes. • It also suggests that Great Occipital Nerve Stimulation, a less invasive procedure, may be effective for treating CCH. • The authors anticipate that advancements in surgical techniques and brain imaging will further refine therapeutic approaches for these patients.	Transient reversible diplopia
Franzini et al. 2009^[27]^	Deep Brain Stimulation of the Posterior Hypothalamus in Chronic Cluster Headache	Prospective Cohort	3	Posterior hypothalamus	• This study discusses the use of DBS in treating CCH targeting the posterior hypothalamus. DBS is well-tolerated and leads to a significant reduction in pain episodes for CCH patients. • Approximately 50–60% of patients respond positively to DBS, allowing many to resume their normal lives and work.	Visual disturbances, primarily diplopia
Franzini et al. 2012^[28]^	Targeting the Brain: Considerations in 332 Consecutive Patients Treated by Deep Brain Stimulation (DBS) for Severe Neurological Diseases	Prospective Cohort	22	Posterior hypothalamus	• Following pHyp-DBS, CCH patients experienced a significant reduction in the intensity and duration of pain bouts, with 71% of postoperative days being pain-free. • The mean time to pain relief was 42 days, and overall drug dosages were reduced to less than 20% of preoperative levels. • At long-term follow-up (8 years), 63% of CCH patients were considered responders to DBS.	No longer benefited in some cases hypomania and suicidal tendencies were the most worrisome adverse effects
Kakusa et al. 2020^[29]^	Electrophysiology and Structural Connectivity of the Posterior Hypothalamic Region: Much to Learn From a Rare Indication of Deep Brain Stimulation	Case Report	1	Left posterior hypothalamus	• DBS in the posterior hypothalamic region effectively treated CH. • The treatment led to long-lasting symptom relief. • Electrophysiological analysis showed changes in brain activity, and structural connectivity analysis revealed the involvement of pain processing regions. • This study supports DBS as a promising therapy for CH and provides insights into its mechanism. • The patient reported lack of headache symptoms and relief of his essential tremor.	Modest recurrence of symptoms but no adverse effects
May et al. 2006 ^[30]^	Hypothalamic Deep Brain Stimulation in Positron Emission Tomography	Prospective Cohort	10	Hypothalamic gray	• Functional imaging has highlighted the hypothalamus’s role in cluster headaches, leading to DBS in this area. • A study with 10 patients used PET scans and alternated DBS on and off. • DBS activated pain processing areas like the thalamus and deactivated regions involved in pain processing. • This suggests that DBS modulates the pain processing network, shedding light on its effectiveness in cluster headache treatment. • All the patients experienced a reduction in headache frequency after hypothalamic stimulation with eight of the 10 patients reporting being completely pain-free while the other two only had sporadic attacks.	No adverse effects reported during the scanning
Nowacki et al. 2019^[31]^	Deep Brain Stimulation of Chronic Cluster Headaches: Posterior Hypothalamus, Ventral Tegmentum, and Beyond	Prospective Cohort	6	Ipsilateral posterior hypothalamic region	• In a study of six chronic cluster headache patients undergoing DBS in the posterior hypothalamic region, long-term results showed a significant reduction in headache severity and improved quality of life. • The stimulating electrodes were located in various brain regions, not just the posterior hypothalamus, suggesting a broader neuroanatomical substrate for DBS-induced headache relief.	Double vision and oscillopsia (reversible)
Starr et al. 2007^[32]^	Chronic Stimulation of the Posterior Hypothalamic Region for Cluster Headache: Technique and 1-Year Results in Four Patients	Case Series	4	Posterior hypothalamus (border zone with anterior PVGM)	• Four patients with medically intractable cluster headaches underwent hypothalamic DBS. • After 1 year, DBS resulted in over a 50% reduction in headache intensity or frequency in two of the four cases. • The active contacts were located just behind the mammillothalamic tract. • While the results are promising, larger trials are needed before widespread clinical use can be recommended.	Intraoperative transient ischemic attack, oculomotor and subjective mood changes at varying voltages
Vetrugno et al. 2007^[﻿33]^	Effect on Sleep of Posterior Hypothalamus Stimulation in Cluster Headache	Case Series	3	The posterior hypothalamus	• DBS of the posterior hypothalamus was evaluated in CCH patients. • Before DBS, patients experienced nocturnal CH attacks and sleep disturbances. • After DBS, CH attacks stopped, sleep improved, and body core temperature rhythm remained normal. T • his suggests that posterior hypothalamus DBS is effective in reducing CH attacks and improving sleep quality without affecting body temperature rhythm.	No adverse effects reported

*CCH *Chronic cluster headaches*, DBS *Deep brain stimulation*, CH *Cluster headaches*, pHyp *Posteromedial hypothalamus

**Table 2:** Comprehensive breakdown of the NIH quality assessment tool

References	Q1	Q2	Q3	Q4	Q5	Q6	Q7	Q8	Q9	Q10	Q11	Q12	Q13	Q14	Overall rating
Franzini 2003	+	+	+	+	NR	+	+	-	+	+	+	NR	+	*	Good
Franzini 2004	+	+	+	+	NA	+	+	+	+	*	+	NR	*	*	Fair
Franzini 2012	+	NR	NA	+	NA	+	+	+	+	+	+	NR	NA	*	Fair
Akram 2017	+	+	+	+	NR	+	+	*	+	+	+	*	+	*	Fair
May 2006	+	+	+	+	NA	+	+	NA	+	NA	+	NR	+	*	Good
Nowacki 2019	+	+	+	+	NR	+	+	NA	+	+	+	-	+	*	Good
P.A.Starr 2007	+	+	+	+	+	+	+	+	+	+	+	NR	+	+	Good
R. Vetrugno 2007	+	+	+	+	-	+	+	+	+	+	+	*	+	*	Good
T. Bartsch 2008	+	+	+	+	+	+	+	+	NR	+	+	*	+	*	Good

Quality Assessment Tool for Observational Cohort and Cross-Sectional Studies (NIH)

Q1: Was the research question or objective in this paper clearly stated?

Q2: Was the study population clearly specified and defined?

Q3: Was the participation rate of eligible persons at least 50%?

Q4: Were all the subjects selected or recruited from the same or similar populations (including the same time period)? Were inclusion and exclusion criteria for being in the study prespecified and applied uniformly to all participants?

Q5: Was a sample size justification, power description, or variance and effect estimates provided?

Q6: For the analyses in this paper, were the exposure(s) of interest measured prior to the outcome(s) being measured?

Q7: Was the timeframe sufficient so that one could reasonably expect to see an association between exposure and outcome if it existed?

Q8: For exposures that can vary in amount or level, did the study examine different levels of the exposure as related to the outcome (e.g., categories of exposure, or exposure measured as continuous variable)?

Q9: Were the exposure measures (independent variables) clearly defined, valid, reliable, and implemented consistently across all study participants?

Q10: Was the exposure(s) assessed more than once over time?

Q11: Were the outcome measures (dependent variables) clearly defined, valid, reliable, and implemented consistently across all study participants?

Q12: Were the outcome assessors blinded to the exposure status of participants?

Q13: Was loss to follow-up after baseline 20% or less?

Q14: Were key potential confounding variables measured and adjusted statistically for their impact on the relationship between exposure(s) and outcome(s)?

(*): Unclear

(+): Yes/Low risk

(-): No/High risk

**Table 3:** Comprehensive Breakdown of the RoB2 Quality Assessment Tool

References	Randomization process	Deviations from Intended Intervention	Missing outcome data	Measurement of the outcome	Selection of the reported result	Overall
Fontaine 2009	+	+	+	+	+	Low Risk
Fontaine 2010	-	*	+	+	+	High Risk

Cochrane Risk of Bias Tool for Randomized Controlled Trials (ROB)

(*): Unclear

(-): No/High risk

(+): Yes/Low risk

### Overall efficacy of DBS for cluster headache

Several studies reported positive outcomes of DBS for CH. These studies found that DBS, particularly targeting the posterior hypothalamus, led to significant reductions in CH ‘attack’ frequency, severity, and duration. The percentage of patients experiencing relief varied, with some studies reporting response rates of approximately 50–60%, while others achieved complete pain remission in a subset of patients. Overall, these findings emphasize the potential of DBS to significantly enhance the quality of life for individuals living with CH, offering a renewed sense of hope and relief from this severe headache disorder. However, further research and refinement of patient selection criteria are essential to optimize the effectiveness of DBS in treating CH.

### Variability in patient response

It is important to note that patient responses to DBS for CH varied. While many patients experienced substantial relief and resumed normal activities of daily life, others showed only marginal improvement or were classified as non-responders. Some studies suggested the existence of different population subtypes within the same diagnostic category, emphasizing the need for patient selection and targeting refinement [17]. These observations highlight the critical role of patient selection and the refinement of targeting strategies in optimizing DBS outcomes for CH. Tailoring treatment to the specific characteristics and needs of individual patients will likely be a key factor in enhancing the overall efficacy of DBS in managing this debilitating condition.

### Safety and tolerability

DBS for CH was generally well-tolerated with limited adverse reactions. Transient, reversible side effects such as diplopia were the most common described in the literature. There were also reports of surgical morbidity, but severe complications were rare. No significant long-term stimulation-related side effects were observed. These findings support the notion that DBS can be a viable therapeutic approach for individuals suffering from CH, with the benefits of symptom reduction outweighing the risks of adverse events [18]. However, it is paramount for healthcare providers to carefully monitor patients and manage any potential adverse effects to ensure the best possible outcomes.

### Anatomical and electrophysiological insights

Anatomical analysis of DBS electrode placement revealed that stimulating contacts were often located in the posterior hypothalamus, red nucleus, mesencephalic grey substance, and other adjacent regions. Electrophysiological recordings exhibited changes in brain activity, including delta and alpha-band power increases near the posterior hypothalamus [21]. Furthermore, there was evidence of functional modulation of the pain processing network, suggesting that **DBS’s** mechanism of action extends beyond direct hypothalamic stimulation. Transduction, modulation, transmission, and perception are the four processes that are known to be involved in the processing of pain-related information. Sensation is produced when a stimulus triggers the activation of the various sensory receptors (transduction), which is then conveyed to the sensory brain (transmission). The combination of precise anatomical targeting, observed changes in brain activity, and evidence of pain network modulation underscores the potential of DBS as a multifaceted treatment option for CH. These insights expand our understanding of how DBS exerts its therapeutic effects beyond localized stimulation. However, further standardized studies and trials with higher sample size designs are needed [22]. 

### Long-term benefits and quality of life improvements

Patients who responded positively to DBS often experienced long-term benefits, with sustained reductions in CH symptoms even after several months to years of follow-up. Quality of life measures consistently showed improvements among responders, suggesting that DBS not only reduces pain but also enhances overall well-being. These findings underscore the potential transformative impact of DBS on the lives of CH patients, offering them the prospect of long-lasting relief from the burdensome symptoms of this condition and an improved quality of life.

### Sleep and circadian rhythm effects

One study investigated the impact of DBS on sleep and the circadian rhythm of body core temperature (BcT°). It found that DBS effectively curtailed nocturnal CH attacks, leading to improved sleep structure and quality [32]. Importantly, the circadian rhythm of BcT° remained normal throughout DBS, indicating no disruption to this critical physiological process. Study results suggested that DBS may alleviate the nocturnal ‘attacks’ observed in CH that often disrupt sleep patterns, precipitating improved sleep quality for CH patients. Importantly, the normalcy of the circadian rhythm of BcT° peri-DBS indicates that this therapeutic approach may provide relief without adversely affecting critical physiology.

## Discussion

We employed a systematic review to perform a complete retrieval of the current literature across several academic databases to explore scientific articles pertaining to the outcomes of utilizing DBS in the treatment of CH. Our systematic review identified several studies regarding both the safety and effectiveness of DBS in the hypothalamus for the treatment of headaches. There were several key findings elucidated from the literature. Several studies indicated positive outcomes when utilizing DBS in the treatment and management of CH. Specifically, DBS often led to a significant reduction in CH ‘attack’ frequency, severity, and duration [20]. However, other studies noted that there was some variability regarding patient responses to DBS for CH [22, 23]. There are probably variations in the effectiveness of DBS for CH, depending on several aspects including the severity of the condition at first and its subtypes [24]. The literature indicated mostly transient, reversible adverse events such as diplopia [25]. DBS may contribute to the functional modification of the pain processing network since it has been demonstrated to have effects that extend beyond the hypothalamus [26]. Patients responding positively to DBS often had sustained positive benefits which is promising to consider that many treatments for CH may diminish in efficacy over time. Lastly, there was a finding that showed DBS curtailed nocturnal CH attacks, thereby leading to improvements in sleep structure and quality [26]. 

Collectively, these findings suggest that DBS may be a viable therapeutic intervention for the management of CH. It may do so through functional modulation of multiple brain structures involved in nociception [28–31]. Despite transient, reversible adverse events the overall benefits (i.e., long-term, and short-term) appear to outweigh the risks. Although DBS may not be an effective treatment option for all patients with CH, it may be another tool that can be introduced as part of an individualized treatment plan [32, 33]. The included, relevant studies had quality that ranged from fair to good and given that none of these studies exhibited poor methodology or were designated as having a high risk of bias, as others we have a good degree of confidence in the findings obtained in these studies.

Headache and confusion—the most frequent implantation-related adverse effects in the early postoperative phase—were temporary [34, 35]. Lead insertion-related symptomatic vascular accidents are the most significant consequence of DBS device installation, and they were rare in our series for both ICH (1.1%) and infarction (0.4%) [36, 37]. There are extremely few reports of cortical or subcortical ischemia infarction associated with DBS in the literature; it happens in roughly one patient per series (0.3–0.9%). This discovery might result from the fact that most ICH patients are asymptomatic and that it is difficult to detect on standard postoperative CT imaging [37]. 

However, there are several limitations in our present study, some of which are inherent to our systematic review methodology. One is that there were a limited number of studies that satisfied our inclusion criteria and were relevant to **DBS** and CH, therefore only 17 were included. This does indeed highlight the need for additional studies and research, especially in areas that provide a better understanding of tailored interventions. Furthermore, it is more likely that the included published studies contained significant findings as publication bias may hinder the publication of studies with non-significant or null associations. There is also subjectivity inherent in the process of a literature review, although we are relatively confident that our methodology minimizes bias by utilizing a strict, well-defined protocol and multiple reviewers.

These studies offer a promising glimpse into the possibility of utilizing DBS in the treatment of CH, especially in individuals who are resistant to or do not have positive outcomes with other treatment methodologies. Future studies should focus on several aspects that are currently unknown and merit further rigorous study. One such avenue is in how DBS exerts therapeutic effects beyond localized hypothalamic stimulation. Another is further exploring how and to what extent DBS can alleviate nocturnal CH attacks.

## Conclusion

DBS shows promise as a treatment for CH. Existing research suggests that DBS, particularly when targeting the posterior hypothalamus, can significantly reduce the frequency, severity, and duration of CH attacks. While individual responses vary, DBS generally offers safety and tolerability, with reversible side effects. DBS may work through a multifaceted mechanism, affecting brain activity and pain processing networks. It also has a positive impact on sleep and circadian rhythms. Overall, DBS has the potential to transform CH treatment, especially for patients unresponsive to other therapies. Future research should focus on refining patient selection and comprehending the foundations of DBS to optimize its efficacy.

## Data Availability

A registered systematic review. Registration on PROSPERO: PROSPERO 2022 CRD42022322666 Available from: https://www.crd.york.ac.uk/prospero/display_record.php? ID=CRD42022322666.

## Abbreviations

DBS: Deep Brain Stimulation
CH: Cluster Headache
CVH: Chronic Venous Hypertension
PET: Positron Emission Tomography
fMRI: functional Magnetic Resonance Imaging
PROSPERO: The International Prospective Register of Systematic Reviews
NA: Not Applicable
NR: Not Reported
NIH: National Institutes of Health
CCH: Chronic Cluster Headache
pHyp: Posteromedial Hypothalamus
BcT°: Body core temperature
